# The Prevalence of Antinuclear Antibodies in Patients with Sarcoidosis

**DOI:** 10.1155/2014/351852

**Published:** 2014-12-17

**Authors:** Senol Kobak, Hatice Yilmaz, Fidan Sever, Arzu Duran, Nazime Sen, Ahmet Karaarslan

**Affiliations:** ^1^Department of Rheumatology, Faculty of Medicine, Sifa University, Bornova, 35100 Izmir, Turkey; ^2^Department of Chest Disease, Faculty of Medicine, Sifa University, Izmir, Turkey; ^3^Department of Microbiology, Faculty of Medicine, Sifa University, Izmir, Turkey; ^4^Department of Orthopedics, Faculty of Medicine, Sifa University, Izmir, Turkey

## Abstract

*Introduction*. Sarcoidosis, which is a chronic inflammatory granulomatous disease, can mimic different rheumatologic diseases including connective tissue diseases. Antinuclear antibodies are the markers used for connective tissue diseases. *Aim*. To determine antinuclear antibody frequency and any possible correlation with clinical and laboratory data in sarcoidosis patients. *Material and Method*. Forty-two sarcoidosis patients, 45 rheumatoid arthritis patients, and 45 healthy volunteers who were followed up in rheumatology outpatient clinic were included in this study. Demographic, clinical, serological, and radiological data of all patients were recorded. Antinuclear antibodies were determined with indirect immunofluorescent method and 1/100 titration was accepted as positive. The cases that were ANA positive were evaluated with immunoblot method. *Results*. Average age of the 42 patients (10 males) with sarcoidosis was 45.2 (20–70 years), and average disease duration was 3.5 years. ANA positivity was detected in 12 (28.5%) patients with sarcoidosis (1/100 in 10 patients, 1/320 in two patients), in 19 of RA patients (42.2%), and in two of healthy volunteers in low titer (*P* < 0.001). In the subgroup analysis made by immunblot test, one patient had anticentromere antibody, one had anti-Ro antibody, one had anti-Scl-70 antibody, one had anti-dsDNA antibody, and eight patients were negative. The two patients who had anticentromere and anti-Scl-70 antibodies had also Sjögren's syndrome and scleroderma diagnosis, respectively. *Discussion*. The prevalence of ANA in patients with sarcoidosis was found to be significantly higher than healthy control group and lower than RA patients. This result shows that ANA may have an important role in the pathogenesis of sarcoidosis and also could be important in revealing the overlap syndromes of sarcoidosis-connective tissue diseases. Further studies with larger series are necessary in this subject.

## 1. Introduction

Sarcoidosis is a systemic disease characterized by the involvement of multiple tissues and organs and a noncaseating granulomatous reaction [1]. Although its pathogenesis is not clear, there appears to be a cellular immune system activation and a nonspecific inflammatory response in genetically predisposed individuals [2]. Proinflammatory cytokines produced by Th1 cells and macrophages trigger the inflammatory cascade, and granuloma formations occur as a result of tissue permeability, cellular influx, and local cell proliferation [3]. The indispensable pathological finding of sarcoidosis is noncaseating epithelioid cellular granulomas [4]. Increased active CD4 T-lymphocytes have been shown in tissues with sarcoidosis. These lymphocytes are shown to increase diffusely in the granulomatous lesion, while small numbers of CD8 T-lymphocytes, B-cells, plasma, and mast cells are identified on the outer part of the granuloma [5]. Sarcoidosis is a chronic granulomatous disease that may present with different clinical findings. It may mimic some manifestations of a number of primary rheumatic diseases and/or develop concomitantly with these. The disease most frequently presents with bilateral hilar lymphadenopathy, infiltrations in the lungs, and skin and eye lesions. Locomotor system involvement is determined to be %15–25 [6]. Two major joint involvement patterns have been identified: acute and chronic forms. Being the most common one, the acute form can be the first sign of sarcoidosis and may present with arthralgia, arthritis, and/or periarthritis. Chronic sarcoid arthritis is usually accompanied by pulmonary parenchymal disease or other organ involvement and occurs rarely and at a late stage of the disease [7]. Serum levels of some biochemical indicators, such as angiotensin converting enzyme (ACE) and calcium, may be elevated in sarcoidosis patients. Clinical, radiological, and laboratory parameters are used to determine the diagnosis of this disease, which can involve multiple systems, and serum indicators are screened accordingly.

Antinuclear antibody (ANA) is the brand name of the antibodies towards nuclear and cytoplasmic structures of the cell. ANA is a screening test used for rheumatologic and nonrheumatologic autoimmune diseases [8]. The most ideal evaluation is immunofluorescent assay (IFA); staining patterns and titers can be determined [9]. ANA positivity with low titers and dilutions may be present in one of three healthy people and these false positivities decrease when the dilution increases [10]. ANA positivity can be present in some of disorders other than connective tissue diseases [11, 12].

Sarcoidosis is a multisystem, inflammatory, chronic disease. It can accompany different connective tissue disorders [1, 13]. It is still discussed if this togetherness is just a coincidence or due to a common etiopathogenesis. Data about ANA positivity in sarcoidosis patients is limited in the literature. Could ANA, being a screening test for connective tissue disorders, have a clinical importance in patients with sarcoidosis?

The purpose of this study is to determine the prevalence of RF, complement (C3 and C4), and antinuclear antibodies and evaluate a possible correlation with clinical and laboratory findings in sarcoidosis patients.

## 2. Material and Method

Forty-two patients presenting to the rheumatology outpatient clinic and diagnosed with sarcoidosis as a result of the examinations made and 45 RA patients diagnosed according to the ACR classification criteria were enrolled in the study. As control group, 45 healthy subjects of matching age and gender were included in the study. Informed consent forms were obtained from all patients. Diagnosis of sarcoidosis was made by demonstration of noncaseating granulomas with biopsies collected from different organs and tissues and histopathological examination. Other possible causes of granulomatous diseases (tuberculosis, bacterial, and fungal infections) were excluded. Laboratory examinations were made for all sarcoidosis patients; routine biochemistry, acute phase reactants (ESR, CRP), serum angiotensin converting enzyme (ACE), serum calcium, and serum 25-hydroxy-vitamin D3 levels were checked. Thorax CT scan was made to stage sarcoidosis. All cases were inquired in a detailed manner; they were given systemic and rheumatic examination. Demographic, clinical, serological, and radiological data of all patients were recorded. Antinuclear antibodies were determined with indirect immunoflorescent method and 1/100 titration was accepted as positive. The cases that were ANA positive were evaluated with immunoblot method. Serum complement (C3 and C4) levels and rheumatoid factor (RF) IgM were determined with the nephelometry method. RF level of >14 IU/mL was considered as significant.

### 2.1. Statistical Analysis

All statistical analyses were made by using SPSS version 9.0 (Chicago, II, USA). Prevalence was calculated for each group and comparisons for categorical variables were made with a Chi-square test. Continuous variables were compared with Student's *t*-test. For all statistical tests, a *P* value of <0.05 was considered to be statistically significant.

## 3. Results

Forty-two sarcoidosis patients (10 males) were included in the study. Mean patient age was 45.2 years (20–70 years) and mean duration of disease was 3.5 years. The system and organ involvement evaluation revealed erythema nodosum in 20 patients (47.6%), uveitis in three patients (7.1%), neurosarcoidosis in one patient (2.3%), and arthritis in 32 patients (76.2%). In patients with arthritis, 28 had ankle joint (87.5%), three had knee joint, and one had wrist joint involvement. None of the patients had cardiac involvement. Thorax CT revealed sarcoidosis stage one in 12 patients (28.5%), stage two in 22 patients (52.4%), stage three in 4 patients (9.5%), and stage 4 in 4 patients (9.5%) (Table 1). Histopathological verification of sarcoidosis was done with endobronchial ultrasound and mediastinoscopy and skin and axillary LAP biopsy revealing noncaseating granuloma. Laboratory tests revealed elevated serum ACE in 15 patients (35.7%), elevated serum calcium in six patients (14.3%), and elevated serum 25-hydroxy-vitamin D3 in two patients (4.7%). The laboratory tests revealed elevated erythrocyte sedimentation rate (normal < 20 mm/h) in 25 (59.5%) patients and elevated C-reactive protein (normal < 5 mg/dL) in 23 (54.7%) patients. Of the 45 patients enrolled in the study with a diagnosis of RA, 12 were males and 33 were females; their mean age was 48.4 years and mean duration of disease was 9.2 years. ANA positivity was detected in total 12 (28.5%) patients with sarcoidosis (10 patients at 1/100, two patients at 1/320 titer) and 19 patients with rheumatoid arthritis (42.2%) and in two of healthy volunteers (*P* < 0.001). The subgroup analysis of 12 patients with immunoblot test revealed anticentromere antibody in one patient, anti-Ro antibody in one patient, anti-Scl-70 antibody in one patient, and anti-dsDNA antibody in one patient, and eight patients were negative. The two patients who had anticentromere and anti-Scl-70 antibodies had also Sjögren's syndrome and scleroderma diagnosis, respectively. RF positivity was detected in 7 sarcoidosis patients (16.6%), in 33RA patients (78.5%), and in only one person in the healthy control group. Three of 42 patients (7.1%) had elevated C4 level and one patient had elevated C3 level (2.3%).

## 4. Discussion

In this study, the prevalence of antinuclear antibodies was found to be significantly higher than healthy control group and lower than RA patients. The two patients found to be positive for anticentromere and anti-Scl-70 antibodies were also diagnosed with Sjögren's syndrome and scleroderma, respectively. RF positivity was detected in seven sarcoidosis patients (16.6%) and in only one person in the healthy control group. Sarcoidosis is a chronic systemic inflammatory disease of unknown etiology, characterized by noncaseating granuloma formations [14]. Clinical, radiographic, and laboratory parameters are used to diagnose this multisystem disease. In some of the patients with sarcoidosis, biochemical markers such as serum ACE, calcium, and 25-hydroxy-vitamin D3 may be elevated in the serum [3]. The elevation of these markers helps us in disease diagnosis. Nevertheless, a specific marker related to sarcoidosis has not been found. Immunogenic abnormalities of sarcoidosis are characterized with Th1 immunological response and accumulation of macrophages in the inflammation area and specifically the lung [4]. In the lesion, an excess cellular immune response towards an unknown agent and different antibodies like RF and ANA may be produced. Increases in serum immunoglobulin, ANA, anti-CCP, RF, and antiphospholipid antibody levels are rare findings and were reported in some previous studies [15–17]. The clinical importance of these antibodies in sarcoidosis is controversial. In our study, ANA, RF, and complement frequencies are evaluated in sarcoidosis patients, where locomotor system involvement is an important finding. Our results showed that ANA positivity was detected in 12 patients (28.5%) and two of these patients coexisted with Sjögren's syndrome and scleroderma also. There are case reports of sarcoidosis and Sjögren's syndrome, and scleroderma togetherness in literature [18, 19]. In a retrospective study, ANA positivity was detected in 10 of 34 sarcoidosis patients diagnosed in 15-year duration. Two patients had anti-ds DNA positivity. All of the 34 patients had normal C3 level. As in our study, in this paper anti-ds DNA antibodies were shown to be present in sarcoidosis patients but not predictable for SLE [20]. Even though no SLE was observed in these 15 years of retrospective study, sarcoidosis patients have to be followed up for a long time for SLE and other connective tissue disorders. In the patients receiving corticosteroid treatment, antibody production may have been suppressed and false negativity may be possible. In these patients and also our patients, steroid and quinine utilization is thought to have masked the symptoms and findings of this disorder.

Rheumatoid factor (RF) is the autoantibody that was first found in rheumatoid arthritis. It is defined as an antibody against the Fc portion of IgG. RF and IgG join to form immune complexes that contribute to the disease process. While RF is usually associated with RA, it may be seen in other diseases and elderly people too. In literature, low-titer RF positivity has been reported in sarcoidosis [21, 22]. In our study, RF positivity in sarcoidosis patients was found to be higher than the control group and lower than RA patients.

Our study has certain limitations. The patient number is low; this prevents having a generalization about the role of ANA in sarcoidosis patients. Using different methods to detect ANA may give an idea about its frequency. However, studying these antibodies in the bronchoalveolar lavage fluid would make a significant contribution to the etiopathogenesis of sarcoidosis.

In conclusion, we have determined that the prevalence of ANA is lower in sarcoidosis patients than RA patients and higher than the control group. Sarcoidosis can mimic many rheumatic diseases. In sarcoidosis cases presenting with serious joint involvement, connective tissue diseases should be watched for in differential diagnosis, and checking ANA levels can be useful in the early diagnosis and treatment of these diseases. Further studies with larger series are necessary in this subject.

## Figures and Tables

**Table 1:** Demographic, clinical, and laboratory features in patients with sarcoidosis.

Features	Patients, *N* = 42 (%)
Age, mean, year	45.2 years
Disease duration, mean, year	3.5 years
Sex (women/men)	32/10
Erythema nodosum	20 (47.6%)
Uveitis	3 (7.1%)
Myositis	1 (2.3%)
Neurosarcoidosis	1 (2.3%)
Arthritis	32 (76.2%)
Elevated serum ACE level (normal 8–52 U/L)	15 (35.7%)
Antinuclear antibody positivity	12 (28.5%)
RF positivity (normal < 14 IU/mL)	7 (16.6%)
Elevated serum C3 (normal 0.9–1.8 g/L)	1 (2.3%)
Elevated serum C4 (normal 0.1–0.4 g/L)	3 (7.1%)
Stage 1 sarcoidosis	12 (28.5%)
Stage 2 sarcoidosis	22 (52.4%)
Stage 3/4 sarcoidosis	4/4 (9.5%)
